# Reversing Multidrug Resistance in Caco-2 by Silencing MDR1, MRP1, MRP2, and BCL-2/BCL-xL Using Liposomal Antisense Oligonucleotides

**DOI:** 10.1371/journal.pone.0090180

**Published:** 2014-03-17

**Authors:** Yu-Li Lo, Yu Liu

**Affiliations:** Department of Biological Sciences and Technology, National University of Tainan, Tainan, Taiwan; Taipei Medical University, Taiwan

## Abstract

Multidrug resistance (MDR) is a major impediment to chemotherapy. In the present study, we designed antisense oligonucleotides (ASOs) against MDR1, MDR-associated protein (MRP)1, MRP2, and/or BCL-2/BCL-xL to reverse MDR transporters and induce apoptosis, respectively. The cationic liposomes (100 nm) composed of N-[1-(2,3-dioleyloxy)propyl]-n,n,n-trimethylammonium chloride and dioleoyl phosphotidylethanolamine core surrounded by a polyethylene glycol (PEG) shell were prepared to carry ASOs and/or epirubicin, an antineoplastic agent. We aimed to simultaneously suppress efflux pumps, provoke apoptosis, and enhance the chemosensitivity of human colon adenocarcinoma Caco-2 cells to epirubicin. We evaluated encapsulation efficiency, particle size, cytotoxicity, intracellular accumulation, mRNA levels, cell cycle distribution, and caspase activity of these formulations. We found that PEGylated liposomal ASOs significantly reduced Caco-2 cell viability and thus intensified epirubicin-mediated apoptosis. These formulations also decreased the MDR1 promoter activity levels and enhanced the intracellular retention of epirubicin in Caco-2 cells. Epirubicin and ASOs in PEGylated liposomes remarkably decreased mRNA expression levels of human MDR1, MRP1, MRP2, and BCL-2. The combined treatments all significantly increased the mRNA expressions of p53 and BAX, and activity levels of caspase-3, -8, and -9. The formulation of epirubicin and ASOs targeting both pump resistance of MDR1, MRP1, and MRP2 and nonpump resistance of BCL-2/BCL-xL demonstrated more superior effect to all the other formulations used in this study. Our results provide a novel insight into the mechanisms by which PEGylated liposomal ASOs against both resistance types act as activators to epirubicin-induced apoptosis through suppressing MDR1, MRP1, and MRP2, as well as triggering intrinsic mitochondrial and extrinsic death receptor pathways. The complicated regulation of MDR highlights the necessity for a multifunctional approach using an effective delivery system, such as PEGylated liposomes, to carry epirubicin and ASOs as a potent nanomedicine for improving the clinical efficacy of chemotherapy.

## Introduction

A major impediment to the success of human cancer therapy is the development of cancer variants exhibiting multidrug resistance (MDR). These variants may develop resistance to drugs with different structures and functions [1]. MDR plays a critical role in tumor initiation and progression by promoting cell proliferation and inhibiting apoptosis [2], [3]. Various mechanisms contribute to MDR, including the overexpression of drug efflux pumps (pump resistance) and the upregulation of cellular antiapoptotic defense systems (nonpump resistance) [4]. P-glycoprotein (P-gp; encoded by *MDR1* gene) and multidrug resistance-associated proteins (MRPs) belong to the ATP-binding cassette (ABC) superfamily. These transporter proteins (responsible for pump resistance) mediate the efflux of drugs in the MDR spectrum, such as anthracyclines, out of cells and thus reduce drug efficacy. Epirubicin (Epi), an epimer of anthracycline doxorubicin, is used for the treatment of breast, gastric, colorectal, and ovarian cancers [5]–[7]. In this study, Epi is selected as a model anticancer drug, because it is a substrate of P-gp, MRP1, and MRP2 [5], [8], [9].

BCL-2 and BCL-xL have been identified as two key inhibitors against various apoptotic stimuli. For cancers in which BCL-2 and BCL-xL are co-expressed, the challenge lies in predicting which antiapoptotic protein is biologically more important for cell survival, and therefore, a more appropriate target for gene therapy. Furthermore, tumor cells often switch expression from BCL-2 to BCL-xL [10]. Collectively, these findings suggest that the simultaneous downregulation of BCL-2 and BCL-xL is a rational strategy for the implementation of anti-cancer therapy [11]. In addition, the literature and our previous investigations have suggested a direct link between the modulation of P-gp and MRPs, and the regulation of apoptosis through BCL-2, BCL-xL, BAX, and caspases [4], [12], [13]. However, the underlying mechanism requires further investigation.

Recent advances in molecular genetics and tumor biology have led to the identification of antisense oligonucleotides (ASOs) for specifically inhibiting the expression of target genes implicated in tumorigenesis and malignant progression [14]–[16]. ASOs are chemically modified nucleotides of single-stranded DNA complementary to the mRNA regions of a target gene. Once introduced into the cell, ASOs hybridize with the RNA complement by Watson–Crick base pairing to form RNA/DNA duplexes, thereby suppressing gene expression effectively and inhibiting protein production [17], [18]. Several ASOs have been used in clinical trials. Fomivirsen, the first antisense drug, was approved by the US Food and Drug Administration in 1998 for ocular cytomegalovirus infection [19]. However, ASOs are mostly polyanionic and hydrophilic. When ASOs are administered into the body, their therapeutic efficacy is low, because they suffer from low transfection efficiency and stability, non-specificity to the target cells, degradation by enzymes, and rapid clearance from the systemic circulation [18], [20]. Polyethylene glycol-coated (PEGylated) cationic liposomes can be successfully used for the intracellular delivery of antineoplastic agents and ASOs directly into the cytoplasm and nuclei of tumor cells to increase their specific anticancer activity and MDR-reversing effect [20], .

**Figure 1:**
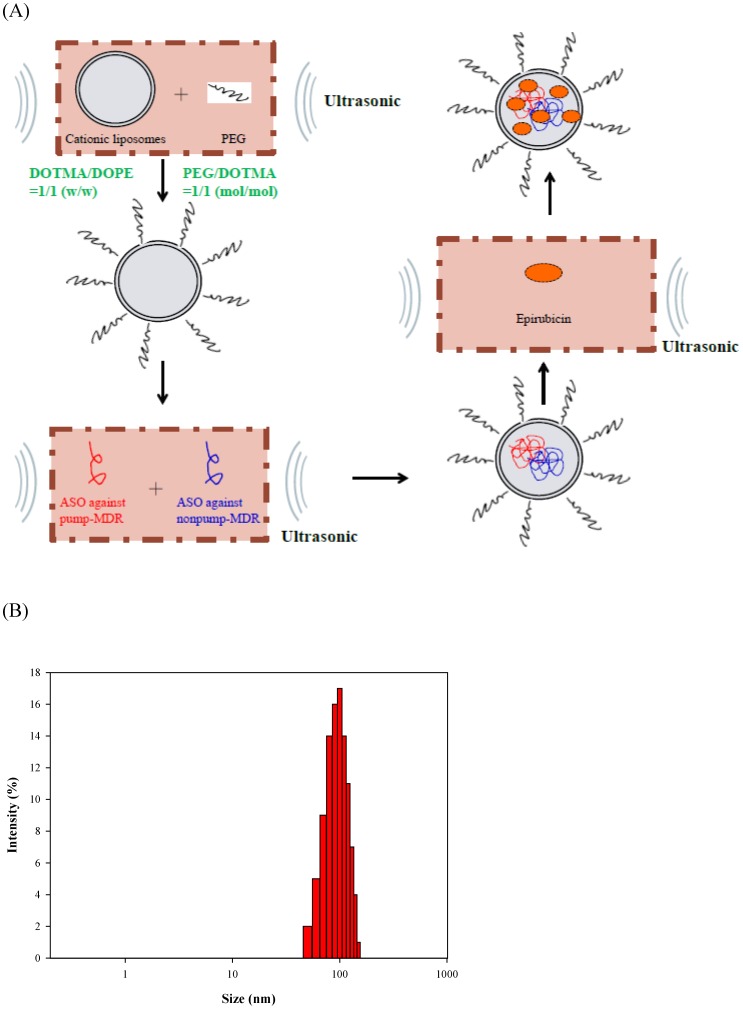
The formation of PEGylated liposomal delivery system of epirubicin and antisense oligonucleotides and particle size distribution of this system. (**A**) A schematic diagram for the formation of PEGylated liposomal delivery system containing epirubicin (Epi) combined with antisense oligonucleotides (ASOs) targeting MDR1, MRP1, MRP2, and/or BCL-2/BCL-xL to reverse pump and nonpump resistances, respectively. (**B**) Particle size distribution of liposomal Epi combined with ASOs targeting MDR1, MRP1, MRP2, and BCL-2/BCL-xL.

In the present study, we proposed a multi-targeted delivery system with four components (Figure 1A): (a) PEGylated cationic liposomes as a delivery system; (b) epirubicin (Epi) as an anticancer drug to trigger apoptosis; (c) ASOs against human MDR1, MRP1, and MRP2 mRNA as an inhibitor of pump resistance; and (d) bispecific ASOs targeting BCL-2 and BCL-xL mRNA as a suppressor of nonpump resistance. The positively charged liposomal core was composed of a cationic lipid, N-[1-(2,3-dioleyloxy)propyl]-n,n,n-trimethylammonium chloride (DOTMA), and a neutral helper lipid, dioleoyl phophotidylethanolamine (DOPE). Steric stabilization was then introduced by incubating preformed liposomes with PEG using the postinsertion method to form a protective shell. Anionic ASOs and/or amphiphilic Epi were encapsulated into the cationic carriers using ultrasonication to reduce the particle size to nanometer scale. We aimed to simultaneously suppress efflux pump proteins, activate apoptosis, and enhance the efficacy of epirubicin on human colon adenocarcinoma Caco-2 cells.

## Materials and Methods

### Reagents

DOTMA and DOPE were purchased from Avanti Polar Lipids, Inc. (Alabaster, AL, USA). Polyethylene glycol 6000 (PEG6000) was obtained from Sigma-Aldrich (St. Louis, MO, USA). Epi was purchased from Pfizer, Inc. (New York, NY, USA). ASOs were obtained from Scientific Biotech Corp. (Taipei, Taiwan). All cell culture medium and reagents were purchased from Promega (Madison, WI, USA), Invitrogen (Carlsbad, CA, USA), Gibco BRL (Grand Island, NY, USA), or Hyclone (Logan, UT, USA). Most of the other chemical reagents were obtained from Sigma-Aldrich (St. Louis, MO, USA) or Merck (Darmstadt, Germany).

### Cell lines

Caco-2 cells were obtained from the Bioresource Collection and Research Center of the Food Industry Research and Development Institute, Hsinchu, Taiwan. Cells were incubated in Dulbecco's modified Eagle's medium (DMEM) supplemented with 10% fetal bovine serum (FBS; Hyclone, Logan, UT, USA), 0.1 mM nonessential amino acids, and 10,000 units/ml of penicillin/streptomycin (Gibco BRL) at 37°C in a humidified atmosphere of 5% CO_2_ and 95% air.

### Preparation of PEGylated cationic liposomal formulations

The sequences of these ASOs are shown in Table I. The sequence of scramble ASOs does not silence or overexpress any known mammalian gene. Based on this design, we have performed treatments of 14 groups (Table II): control (CTR); scramble ASOs (SCR); free Epi; ASOs against MDR1, MRP1, and MRP2 (ASOs against pump resistance); ASOs against BCL-2/BCL-xL (ASOs against nonpump resistance); ASOs against MDR1, MRP1, MRP2, and BCL-2/BCL-xL (ASOs against both resistances); empty liposome (Lip); liposomal Epi (Lip-Epi); liposomal ASOs against MDR1, MRP1, and MRP2 (Lip-ASOs against pump resistance); liposomal ASOs against BCL-2/BCL-xL (Lip-ASOs against nonpump resistance); liposomal ASOs against MDR1, MRP1, MRP2, and BCL-2/BCL-xL (Lip-ASOs against both resistances); liposomal Epi plus ASOs against MDR1, MRP1, and MRP2 (Lip-Epi+ASOs against pump resistance); liposomal Epi plus ASOs against BCL-2/BCL-xL (Lip-Epi+ASOs against nonpump resistance); and liposomal Epi plus ASOs against MDR1, MRP1, MRP2, and BCL-2/BCL-xL (Lip-Epi+ASOs against both resistances).

**Table 1:** Sequences of antisense oligonucleotides.

Category of antisense oligonucleotides (ASOs)^a^	Sequences
ASOs against MDR1^b^	5′-TTC AAG ATC CAT CCC GAC CTC GCG-3′
ASOs against MRP1^c^	5′-TGC TGT TCG TGC CCC CGC CG-3′
ASOs against MRP2^d^	5′-GGC TGC CAT GGT CCC-3′
Bispecific ASOs against BCL-2 and BCL-xL	5′-AAG GCA TCC CAG CCT CCG TT-3′
Scramble ASOs	5′-AATTCTCCGAACGTGTCACGT-3′

a The DNA backbone of all bases in oligonucleotides is P-ethoxy modified to enhance nuclease resistance and increase incorporation efficacy into liposomes.

b MDR1, multidrug resistance gene 1.

c MRP1, gene of MDR-associated protein 1.

d MRP2, gene of MDR-associated protein 2.

**Table 2:** Composition of different formulations.

	CTR	SCR	Epi	ASOs against pump resistance	ASOs against nonpump resistance	ASOs against both resistances	Lip	Lip-Epi	Lip-ASOs against pump resistance	Lip-ASOs against nonpump resistance	Lip-ASOs against both resistances	Lip-Epi+ASOs against pump resistance	Lip-Epi+ASOs against nonpump resistance	Lip-Epi+ASOs against both resistances
Epirubicin (Epi)	**−** a	**−**	+^b^	**−**	**−**	**−**	**−**	+	**−**	**−**	**−**	+	+	+
PEGylated liposomes (Lip)	**−**	**−**	**−**	**−**	**−**	**−**	+	+	+	+	+	+	+	+
ASOs against MDR1	**−**	**−**	**−**	+	**−**	+	**−**	**−**	+	**−**	+	+	**−**	+
ASOs against MRP1	**−**	**−**	**−**	+	**−**	+	**−**	**−**	+	**−**	+	+	**−**	+
ASOs against MRP2	**−**	**−**	**−**	+	**−**	+	**−**	**−**	+	**−**	+	+	**−**	+
ASOs against BCL-2/BCL-xL	**−**	**−**	**−**	**−**	+	+	**−**	**−**	**−**	+	+	**−**	+	+
Scramble ASOs	**−**	+	**−**	+	+	**−**	**−**	**−**	+	+	**−**	+	+	**−**

a **−**: absence of the indicated reagent.

b +: presence of the indicated reagent.

The method of preparation was modified from Li and Huang [21]. Small PEGylated cationic unilamellar liposomes consisting of DOTMA and DOPE (1∶1 w/w) were prepared by thin film hydration. Briefly, DOTMA and DOPE (1 mg/ml) were heated to around lipid phase transition temperature (about 60°C), and then mixed with PEG6000 (1∶1 molar ratio) [22]. This mixture was incubated at 60°C water bath for 2 h, followed by ultrasonication at 25°C for 10 min to ensure the coating of the PEG chains to the liposomal core. Individual ASOs were mixed by ultrasonication at 25°C for 90 min. Epi (200 µg/ml) was incorporated into the PEGylated liposomes (Epi/lipid = 1.6 w/w) to form the PEGylated liposomal Epi and/or ASOs. The final concentrations of Epi and each ASO were 1 µg/ml and 0.22 µM, respectively. For example, Lip-Epi+ASOs against both resistances possessed a final concentration of Epi (1 µg/ml) and each ASOs against MDR1 (0.22 µM), MRP1 (0.22 µM), MRP2 (0.22 µM), and BCL-2/BCL-xL (0.22 µM) with a total ASOs concentration of 0.88 µM. Lip-ASOs against pump resistance contained an individual concentration of 0.22 µM for each ASOs targeting MDR1, MRP1, and MRP2 (total 0.66 µM) and scramble ASOs (0.22 µM) to keep the equally total ASO concentrations of 0.88 µM. This preparation was vibrated by ultrasonication at 25°C for 60 min.

### Encapsulation efficiency (EE%), size distribution, and zeta potential

Unbound Epi and ASOs were separated from the loaded PEGylated liposomes by filtration and centrifugation at 4000× g for 20 min (4°C) through an Amicron Ultra-4 Centrifuge Filter (10,000 WCO, Millipore Corp., Billerica, MA). ASOs in the filtrate were measured by NanoDrop 2000 (Thermo, Wilmington, DE, USA). Epi in the filtrate was analyzed by HPLC.[23] The HPLC system is composed of a L7100 pump (Hitachi, Tokyo, Japan) equipped with an L2200 automated injector, a LiChrospher column (25 cm long, 4 mm inside diameter; Merck), and a L2400 UV detector (Hitachi). The mobile phase was prepared by methanol and water (75∶25, v/v). The flow rate was set at 1.2 ml/min and the detection wavelength was 254 nm. EE1% was calculated as the percentage of the amount of ASOs in liposomes divided by the total amount of added ASOs. EE2% was calculated as the percentage of the amount of Epi encapsulated into liposomes divided by the total amount of added Epi. Each experiment was performed in quadruplicate. EE1% and EE2% were calculated by the equation 1 as shown below.
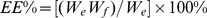
where W_e_ is the weight of added Epi (or ASOs) and W_f_ is the weight of Epi (or ASOs) in the filtrate.

In addition, the size distribution and zeta potential of liposomes were measured using a Zetasizer 3000 HS dynamic light scattering system (Malvern Instruments Ltd., Malvern, Worcestershire, UK). Before measurement, the liposomes were diluted in culture medium and measurement was performed at 25°C. Data was calculated by a cumulant method to obtain polydispersity index. Data were analyzed from four individual measurements and the mean value was calculated.

### Cytotoxicity assay

Cells (6×10^3^) were incubated in 96-well plates and treated with the individual 14 groups (Table II) for 48 h. The cells were mixed with 0.2 mg/ml MTT ((3-(4,5-dimethylthiazol-2-yl)-2,5-diphenyltetrazolium bromide; Sigma) and incubated for another 4 h. Dimethylsulfoxide (DMSO, 100 µl) was added to each well to dissolve the formazan. We detected the optical density value (OD_540_) using an MRX microplate reader (Dynatech Laboratories Inc., Chantilly, VA, USA), which set the wavelength at 540 nm. The measured OD_540_ for different treatments was converted into the cell number according to the standard curve. Relative cell viability (%) was calculated by dividing the number of cells treated with each group by the number of cell control. Data were analyzed from six individual measurements.

### RNA extraction and quantitative real-time PCR of MDR1, MRP1, MRP2, BCL-2, BAX, caspases, and p53

4×10^5^ cells were maintained in 6-well plates and pretreated with control or various formulations for 48 h (Table II) at a final concentration of 1 µg/ml for Epi and/or 0.22 µM for each ASO. After treatment, we used the Total RNA Miniprep System (Viogene, Taipei, Taiwan) to isolate RNA from the cells. RNA yield and purity were evaluated using NanoDrop 2000 (Thermo, Wilmington, DE, USA). cDNA was reverse transcribed from total RNA using a high-capacity RNA-to-cDNA kit (Applied Biosystems; Foster City, CA, USA). Gene-specific primers (Table III) of MDR1, MRP1, and MRP2 (efflux transporter-related), as well as BCL-2, BAX, caspase-3, -8, -9, and p53 (apoptosis-related) were verified by melting curve and agarose gel analyses. GAPDH was used as an internal control. Real-time PCR was performed using the StepOne Real-Time PCR system (Applied Biosystems) and SYBR Green PCR Master Mix (Applied Biosystems). The cycling program was set as follows: denaturation at 95°C for 10 min, followed by 40 cycles of 95°C for 15 s and 60°C for 1 min. The result was assayed from triplicate measurements and normalized to the GAPDH level. The expression ratio of mRNA was calculated as the ratio of the treatment group compared with the cell control.

**Table 3:** Real-time PCR primer sequences used for screen of intestinal MDR transporter-related genes and apoptosis-associated genes.

Gene name	Forward primer	Reverse primer
GAPDH	ATGGGGAAGGTGAAGGTCG	GGGGTCATTGATGGCAACAATA
MDR1	GCTCATCGTTTGTCTACAGTTCGT	ACAATGACTCCATCATCGAAACC
MRP1	GGATCATGCTCACTTTCTGG	AAGTGATGTCACGAAACAGGTC
MRP2	AAGATGCAGCCTCCATAACCA	TGGACCTAGAACTGCGGCTAA
p53	GAGAATCTCCGCAAGAAAGG	CTCATTCAGCTCTCGGAACA
Bcl-2	CTTGACAGAGGATCATGCTGTAC	GGATGCTTTATTTCATGAGGC
Bax	GGGCCCACCAGCTCTGA	CCTGCTCGATCCTGGATGA
Caspase-3	CCTGGTTATTATTCTTGGCGAAA	GCACAAAGCGACTGGATGAA
Caspase-8	CAGGCAGGGCTCAAATTTCT	TCTGCTCACTTCTTCTGAAATCTGA
Caspase-9	TGCTGAGCAGCGAGCTGTT	AGCCTGCCCGCTGGAT

### Plasmid construction of *hMDR1* promoter fragment

The construction of human *MDR1* (*hMDR*1) promoter region has been described in details in our previous studies [9], [12]. Briefly, we amplified the 159-bp *hMDR*1 promoter element of residue −120 to +39 using PCR with primers composed of the 5′-primer (5′-CGCAGTCTCTCGAGCAATCAGCATTAGTCAGTGC) and the 3′-primer (5′-GTCAAGCTTGAGCTTGTAAGAGCCGCTACTAGA). The resulting PCR fragment was transferred into the pGL3-basic firefly luciferase reporter vectors (Promega, Madison, WI, USA) using a T4 DNA ligase and the restriction enzymes *Xho*I and *Hind*III (Promega). All the plasmid products were amplified in *Escherichia coli* competent cells and then purified using a MagneSil Magnetic Separation Unit (Promega).

### Transfection and dual luciferase activity assay

2 µg/well of the *hMDR1* promoter-pGL3 reporter vector constructs were gently mixed with 0.2 µg/well of the pRL-TK *Renilla* luciferase reporter gene (Promega) and 6 µl of Lipofectamine 2000 (Invitrogen Corp., Carlsbad, CA, USA). The mixture was subjected for Caco-2 cell transfection at 25°C for 15 min and the transfected cells were incubated at 37°C for 15 h. Subsequently, the cells were incubated with different treatments for 48 h. 20 µl lysate supernatants were added with 100 µl Luciferase Assay Reagent II (Promega) to start the luciferase reaction. We measured the luciferin luminescence using a luminometer (MiniLumat LB9506; Berthold, Bad Wildbad, Germany). 100 µl Stop & Glo reagent (Promega) was added to simultaneously quench the firefly reaction and initiate a *Renilla* luciferase reaction. After correcting a background luminescence, we computed the data as the *hMDR1* promoter activity level of treatment group divided by that of control.

### Intracellular accumulation of Epi

Cells (2×10^4^) were seeded into 24-well plates. After pretreatment with 14 different formulations at 37°C for 48 h, the cells were harvested in the dark and washed twice by PBS, and intracellular Epi fluorescence was detected using a flow cytometer (Cell Lab Quanta SC MPL; Beckman Coulter, Fullerton, CA, USA) supplied with an argon ion laser and excited at 488 nm. Red Epi fluorescence was evaluated at 575/50 nm (FL-2) after logarithmic amplification. Data measurement and calculation were assessed using commercial software (Cell Lab Quanta SC MPL). At least 10,000 cells were determined in each sample. Four individual experiments were done.

### Caspases-3, -8, and -9 activity assay

Commercially available Caspase-Glo 3, Caspase-Glo 8, and Caspase-Glo 9 Assay Kits (Promega, Madison, WI, USA) were used to measure the caspases-3, -8, and -9 activities. 2×10^5^ cells/well were harvested after different treatments for 48 h. Fifty µl of caspase-3, -8, and -9 substrate reagents were then mixed with 50 µl of the cell suspension at room temperature for 30 min [9]. Released aminoluciferin luminescence levels were detected using a luminometer (MiniLumat LB9506; Berthold, Bad Wildbad, Germany).

### Cell cycle analysis

Apoptotic cells in different cell cycle phases were determined by flow cytometry. Cells (1×10^5^) were seeded into 24-well plates and treated for 48 h. The cells were harvested and mixed with 80% ethanol at −20°C overnight. The cells were then resuspended in hypotonic buffer. We used propidium iodide (1 mg/ml) to stain the cells and incubated them for 30 min in the dark. Data acquisition was achieved using a flow cytometer (Cell Lab Quanta SC MPL). Three individual experiments were performed and analyzed.

### DNA fragmentation

2×10^5^ cells/well were treated with 14 formulations for 48 h. These cells were harvested by centrifugation and the cell suspensions were mixed with lysis buffer, followed by incubation at 56°C for 24 h. Equal volume of phenol, chloroform, and isoamyl alcohol (25∶24∶1) was added to extract the DNA. The DNA product was then separated by 2% agarose gel electrophoresis at 50 V. The resolved gel was visualized using a SYBR® Safe dye (Invitrogen) and digitally scanned using a gel documentation system (UVIdoc; UVItec Limited, Cambridge, UK). Three individual experiments were performed and only one representative electrophoresis plot was shown.

### Statistical analysis

Statistical analysis was done using Student's *t* test and expressed as mean ± standard deviation (S.D.). For multiple group comparison, one-way ANOVA and Dunnett's tests were performed. Differences between groups were set at *P*<0.05.

## Results

### Determination of encapsulation efficiency, particle size, and zeta potential of PEGylated liposomal ASOs or Epi

The encapsulation efficiency (%) of ASOs (EE1%) and Epi (EE2%) in PEGylated liposomes ranged from 86.28±3.50% for Lip-Epi+ASOs against both resistances to 87.97±1.56% for Lip-Epi, as demonstrated in Table IV.

**Table 4:** Physicochemical properties of different liposomal formulations (n = 4).

	Particle size (nm)	Zeta potential (mV)	Polydispersity index	EE1%^a^	EE2%^b^
Lip	95.3±2.8	22.4±2.26	0.091±0.010	**-**	**-**
Lip-Epi	104.3±2.7	26.13±3.43	0.111±0.021	**-**	87.97±1.56
Lip-ASOs against pump resistance	100.8±2.4	16.23±2.29	0.115±0.027	87.84±2.37	**-**
Lip-ASOs against nonpump resistance	103.8±1.3	20.66±2.28	0.099±0.018	87.71±1.49	**-**
Lip-ASOs against both resistances	110.2±1.5	14.04±1.27	0.119±0.023	87.29±1.32	**-**
Lip-Epi+ASOs against pump resistance	105.1±3.5	20.47±2.21	0.109±0.015	86.61±1.47	87.28±1.55
Lip-Epi+ASOs against nonpump resistance	102.3±2.3	24.88±3.22	0.113±0.020	87.16±2.81	86.69±2.38
Lip-Epi+ASOs against both resistances	97.7±1.4	18.34±1.28	0.098±0.022	86.28±3.50	86.31±1.28

a encapsulation efficiency 1 (EE1%) was calculated as the percentage of the amount of ASOs in liposomes divided by the total amount of added ASOs.

b encapsulation efficiency 2 (EE2%) was calculated as the percentage of the amount of Epi in liposomes divided by the total amount of added Epi.

These PEGylated liposomal preparations were well-dispersed particles with sizes ranging from 95.3±2.8 nm for Lip to 110.2±1.5 nm for Lip-ASOs against both resistances, with an acceptably homogeneous polydispersity index about 0.1 (Table IV). The particle size distribution of Lip-Epi+ASOs against both resistances is shown in Figure 1B.

In our prepared liposomes, the mean zeta potential of Lip was 22.4±2.26 mV (n = 4), indicating highly positively charges in this nanoparticle formulation (Table IV). As Epi was encapsulated in Lip, the zeta potential of Lip-E was slightly increased, because of the cationic nature of Epi. When ASOs was incorporated into Lip, the zeta potential of these formulations decreased, probably due to the negative charges of ASOs. The net positive zeta potential in the liposomal formulations containing both Epi and different ASOs may improve their electrostatic interactions with negatively charged surface of cancer cells.

### PEGylated liposomal Epi and ASOs significantly increased Epi cytotoxicity

**Figure 2:**
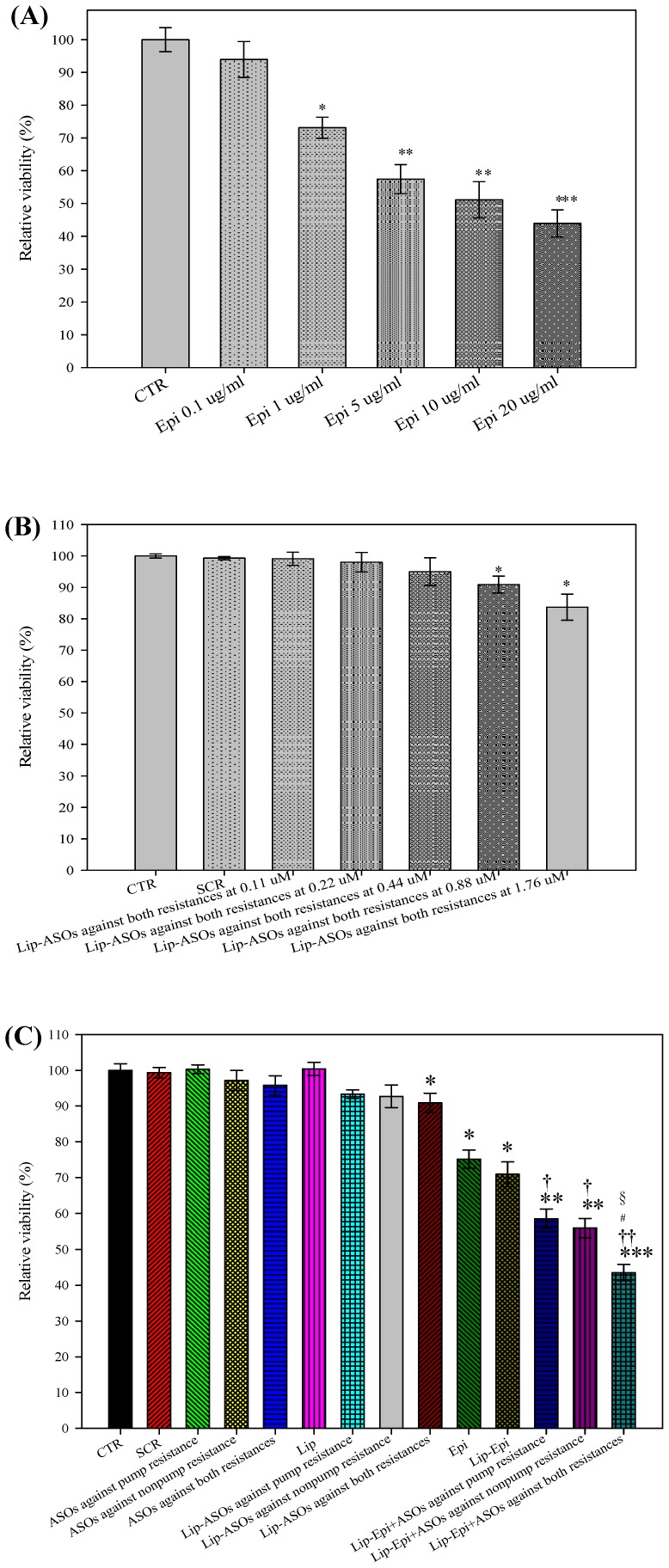
Susceptibility of Caco-2 cells after epirubicin and/or antisense oligonucleotide treatment. (**A**) The effect of Epi at concentrations 0, 0.1, 1, 5, 10, and 20 µg/ml for 48 h incubation on the cell viability of Caco-2 cells. ^*^ *P*<0.05, ^**^ *P*<0.01, and ^***^ *P*<0.001 compared to control (CTR). (**B**) The effect of Lip-ASOs against both resistance types at concentrations of 0, 0.1, 0.22, 0.44, 0.88, and 1.76 µM and 48 h of incubation on the cell viability of Caco-2 cells. ^*^ *P*<0.05 compared to CTR. (**C**) The effect of Epi (1 µg/ml) and ASOs (0.88 µM) in free or liposomal formulations on the cell viability of Caco-2 cells. Means ± S.D. from six independent experiments are shown. ^*^ *P*<0.05, ^**^ *P*<0.01, and ^***^ *P*<0.001 compared to CTR; ^†^ *P*<0.05 and ^††^ *P*<0.01 compared to Epi; ^#^ *P*<0.05 when Lip-Epi+ASOs against both resistances compared to Lip-Epi+ASOs against pump resistance; ^§^ *P*<0.05 when Lip-Epi+ASOs against both resistances compared to Lip-Epi+ASOs against nonpump resistance.

The relative cell viability (%) of cells treated with various concentrations of Epi (0, 0.1, 1, 5, 10, and 20 µg/ml) is shown in Figure 2A. The concentration of Epi necessary to inhibit proliferation or increase death of Caco-2 cells by 50% is expressed as IC_50_. The mean IC_50_ value for the treatment of Epi was 13.95±0.26 µg/ml. ASOs were used as adjuvants to potentiate the cytotoxicity of Epi, and thus 1 µg/ml Epi (<IC_50_) was selected for the following combined treatment with ASOs. The relative viability (%) of cells treated with various concentrations of Lip-ASOs against both resistances (0, 0.11, 0.22, 0.88, and 1.76 µM) is shown in Figure 2B. The concentration of Lip-ASOs against both resistances from 0.11 to 0.44 µM had no significant cytotoxicity to Caco-2 cells. We chose 0.88 µM Lip-ASOs against both resistances (relative cell viability % of 90.89±1.20) to intensify the potency of Epi. As exhibited in Figure 2C, the combined treatment of PEGylated liposomal Epi and ASOs against pump resistance (*P*<0.05), nonpump resistance (*P*<0.05), or both resistances (*P*<0.01) demonstrated more cytotoxicity to Caco-2 cells compared with those of free and liposomal Epi. Lip-Epi+ASOs against both resistances was the most effective formulation to reduce Caco-2 cell viability among all the formulations (all *P* at least <0.05). The relative viability (%) of Lip-Epi+ASOs against both resistances was compatible with the value of free epirubicin at 20 µg/ml. Thus, the combination of 1 µg/ml Epi and 0.88 µM ASOs against both resistances increased the efficacy of Epi to a level of 20-fold (20 µg/ml in monotherapy vs. 1 µg/ml in combined therapy).

### PEGylated liposomal ASOs decreased the mRNA levels of MDR transporters

**Figure 3:**
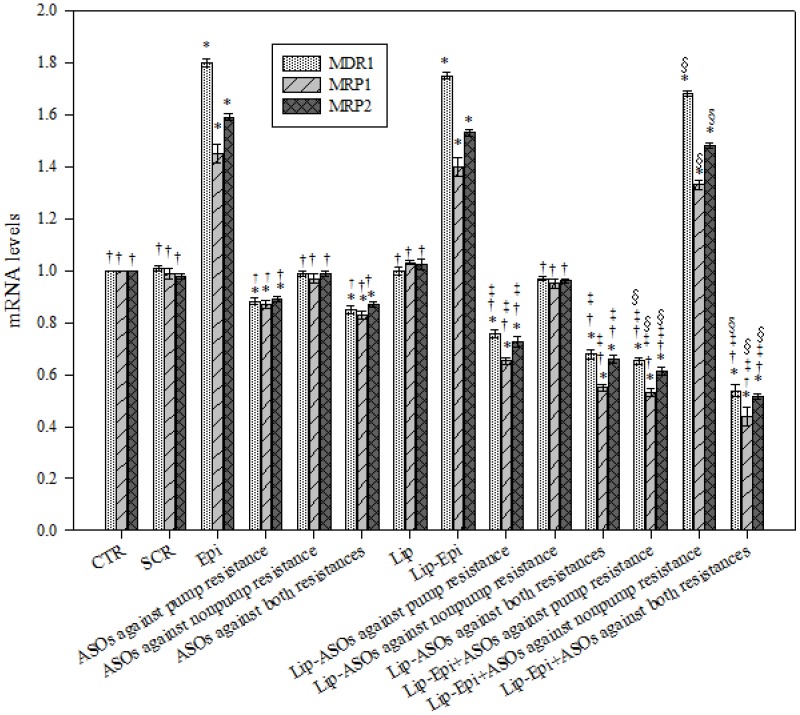
The effect of different treatments on the mRNA levels of pump resistance associated genes. The effect of different treatments on the mRNA levels of MDR1, MRP1, and MRP2, as measured by real-time PCR. Means ± S.D. from three independent experiments are shown. ^*^ *P*<0.05 compared to CTR; ^†^ *P*<0.05 compared to Epi; ^‡^ *P*<0.05 when liposomal formulation compared to its corresponding free formulation. For example, Lip-ASOs against pump resistance vs ASOs against pump resistance. ^§^ *P*<0.05 when liposomal formulation of Epi plus ASOs compared to its corresponding liposomal formulation of ASOs. For example, Lip-Epi+ASOs against both resistances vs Lip-ASOs against both resistances.

The mRNA expression levels of MDR1, MRP1, and MRP2 were evaluated using real-time PCR. Treatments with ASOs against pump resistance, ASOs against both resistances in free or liposomal formulations all significantly decreased the corresponding mRNA levels of MDR1, MRP1, and MRP2 (*P*<0.05, Figure 3). Epi, Lip-Epi, and Lip-Epi+ASOs against nonpump resistance all remarkably increased the mRNA levels of MDR1, MRP1, and MRP2 (*P*<0.05, Figure 3), implying the role of Epi in inducing resistance-related proteins to pump Epi out and exacerbating MDR development. ASOs targeting nonpump resistance in free or liposomal formulations exhibited no significant effect on the inhibition of pump resistance genes (*P*>0.05). Lip-ASOs against pump resistance and Lip-ASOs against both resistances showed significantly greater inhibitory effects on MDR1, MRP1, and MRP2 expressions than those of free formulations, respectively.

### PEGylated liposomal ASOs significantly decreased the luciferase activity of the *hMDR1* promoter region

**Figure 4:**
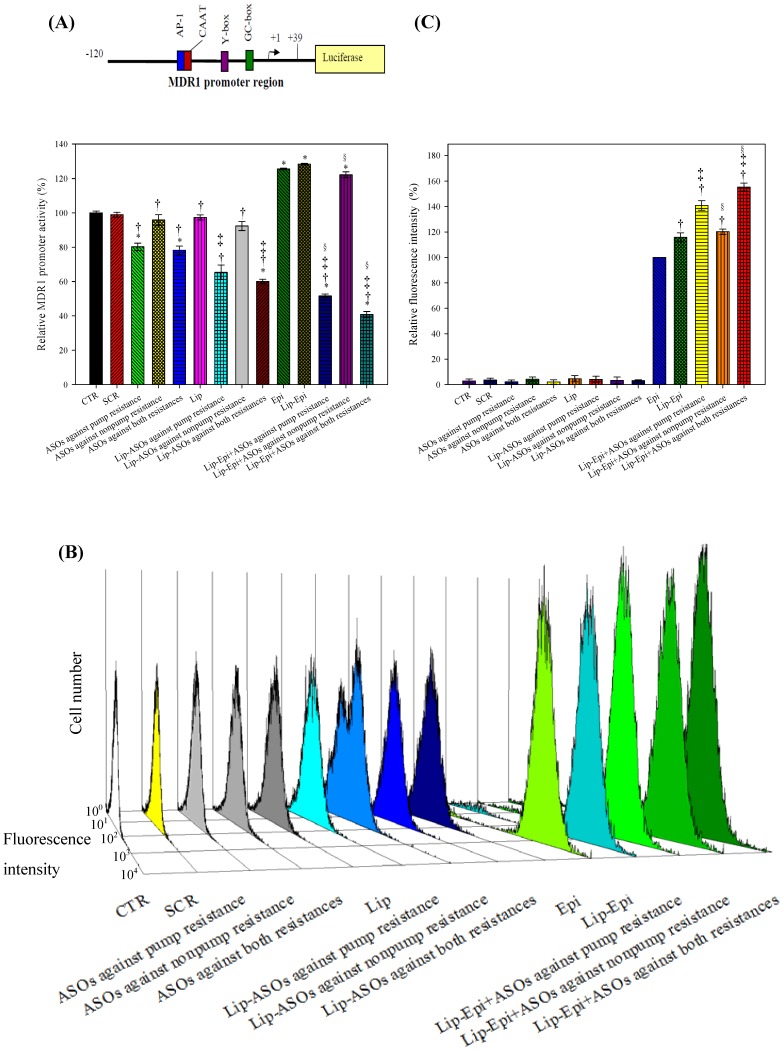
The effect of different treatments on the *hMDR1* promoter activity and the intracellular accumulation of epirubicin in Caco-2 cells. (**A**) The effect of different treatments for 48 h on the *hMDR1* promoter activity in Caco-2 cells. The luminescence was measured using a dual luciferase assay kit with a luminometer. After background correction, results were expressed as the level of *hMDR1* promoter-pGL3 activity divided by pRL-TK activity level. ^*^ *P*<0.05 compared to CTR; ^†^ *P*<0.05 compared to Epi; ^‡^ *P*<0.05 when liposomal formulation compared to its corresponding free formulation; ^§^ *P*<0.05 when liposomal formulation of Epi plus ASOs compared to its corresponding liposomal formulation of ASO. (**B**) The effect of different treatments on the intracellular accumulation of fluorescent Epi in Caco-2 cells. The three-dimensional view of cell number versus fluorescence intensity of Caco-2 cells after different treatments was shown. (**C**) Mean fluorescence intensity of Epi control was normalized to be 100%. Mean fluorescence intensity of the other treatments was normalized relative to the Epi. Data are means ± standard deviation of four independent experiments. The relative fluorescence intensity % of formulations without Epi demonstrates that the auto-fluorescence of these treatments was negligible. ^†^ *P*<0.05 compared to Epi; ^‡^ *P*<0.05 compared to Lip-Epi; ^§^ *P*<0.05 compared to Lip-Epi+ASOs against pump resistance.

To verify if PEGylated liposomal ASOs and/or Epi affected the transcriptional regulation of *hMDR*1 in Caco-2 cells, the promoter elements of 159 bp including a distinct GC box, an inverted CCAAT box (Y box), a CAAT site, and an AP-1 site were transfected into the region upstream of the firefly luciferase reporter gene in the pGL3-basic vector. We selected this promoter region because MDR1 gene transcription is regulated by multiple transcription factors [24]–[26]. ASOs against pump resistance, ASOs against both resistances, their corresponding liposomal formulations, and their combined treatments with Epi significantly decreased *hMDR1* promoter activity levels (*P*<0.05; Figure 4A). Epi, Lip-Epi, and Lip-Epi+ASOs against nonpump resistance significantly increased the luciferase activity (*P*<0.05), which indicated that Epi had remarkably enhanced *hMDR1* promoter activity. In a negative control experiment, we also found that ASOs and/or Epi did not change the luminescence of the *hMDR1* promoter-deficient pGL3 vectors, which implies that there was no nonspecific direct interaction between the individual testing agents and the luciferase reporter genes.

### PEGylated liposomal ASOs increased the Epi retention in Caco-2 cells

Our experimental data (Figure 4B and C) showed that PEGylated liposomal Epi and ASOs against pump resistance enhanced the intracellular accumulation of Epi and Lip-Epi (*P*<0.05) after 48 h treatment. The combined treatment of Epi and ASO targeting nonpump resistance exhibited no further enhancement of Epi retention than Lip-Epi did (*P*<0.05). Lip-Epi+ASOs against both resistance types demonstrated no more retention of Epi than that of Lip-Epi+ASOs against pump resistance (*P*>0.05). These results suggest that inhibiting P-gp and MRPs using specific ASOs may account for the decrease in MDR transporter function and the corresponding increase in Epi's retention in Caco-2 cells.

### Liposomal ASOs and Epi modulated mRNA expressions of apoptosis-related genes

**Figure 5:**
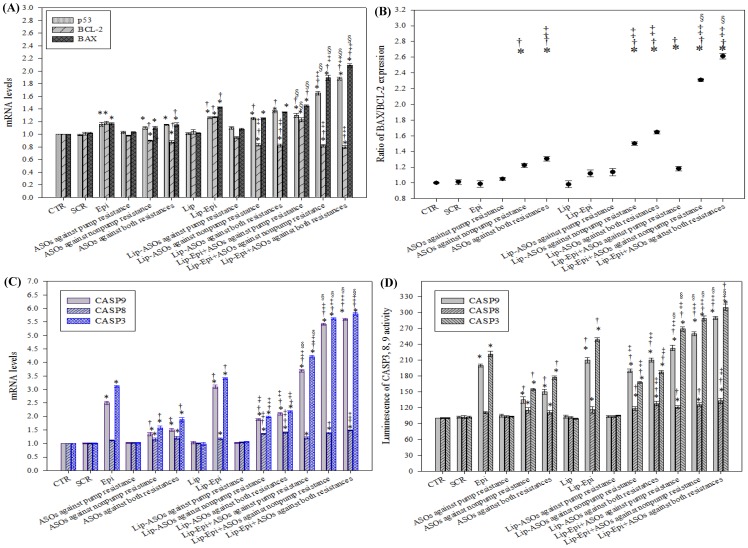
The effect of different treatments on the mRNA levels of nonpump resistance associated genes and caspase activities. (**A**) The effect of different treatments on the mRNA levels of nonpump resistance associated genes, including BCL-2, BAX, and p53, as measured by real-time PCR. (**B**) The BAX:BCL-2 expression ratios of different treatments. (**C**) The effect of different treatments on the mRNA levels of apoptotic genes, including caspase 9 (CASP9), caspase 8 (CASP8), and caspase 3 (CASP3), as measured by real-time PCR. (**D**) The effect of different treatments on CASP3, 8, and 9 activities as recorded the luminescence using a luminometer. Means ± S.D. from three independent experiments are shown. ^*^ *P*<0.05 compared to CTR; ^†^ *P*<0.05 compared to Epi; ^‡^ *P*<0.05 when liposomal formulation compared to its corresponding free formulation; ^§^ *P*<0.05 when liposomal formulation of Epi plus ASOs compared to its corresponding liposomal formulation of ASO.

The mRNA expression levels of BAX, BCL-2, caspase-3, -8, -9, and p53 were evaluated using real-time PCR. The treatments of Lip-Epi plus ASOs against pump resistance, nonpump resistance, or both resistance types significantly increased the corresponding mRNA levels of p53, BAX, caspase-3, -8, and -9 (Figure 5A and C; *P*<0.05), and significantly increased the BAX-to-BCL-2 ratio (Figure 5B; *P*<0.05). Epi and Lip-Epi had a marginal effect on caspase 8, whereas they increased the p53, BAX, caspase-3, and -9 expressions. For the above individual treatments, the changes in the expression level of caspase 8 were much lower than the corresponding caspase 9 (Figure 5C; All *P*<0.05). Lip-Epi+ASOs against both resistances resulted in significantly (*P*<0.05) greater upregulation of p53, BAX, caspase-3, -8, and -9 expressions than all the other treatments (all with *P*<0.05). Interestingly, Epi, Lip-Epi, and Lip-Epi+ASOs against pump resistance induced BCL-2 expression, whereas Lip-ASOs against nonpump resistance, Lip-ASOs against both resistances, and their combined treatments with Epi inhibited BCL-2 expression (All *P*<0.05). Lip-Epi+ASOs against both resistances did not decrease the mRNA levels of BCL-2 more than Lip-Epi+ASOs against nonpump resistance did (*P*>0.05). Whether cells undergo apoptosis or not depends on the dynamic equilibrium of the expression of BAX and BCL-2. The higher BAX/BCL-2 ratio means more cells possess the potential to undergo apoptosis, as shown in Figure 5B. The formulations with Epi as the component significantly increased the BAX/BCL-2 ratio (*P*<0.05). The addition of ASOs in the formulations further enhanced BAX/BCL-2 ratio (*P*<0.05).

### Treatments of liposomal Epi and/or ASOs significantly increased caspases-3, -8, and -9 activities

We examined the activities of caspases-3, -8, and -9 induced by ASOs with or without Epi to determine the apoptotic pathway involved. The activities of these caspases were increased by Epi or ASOs targeting BCL-2/BCL-xL or both resistance types in free or liposomal formulations (*P*<0.05) and further enhanced by the combined treatment (Figure 5D; *P*<0.05). However, the increased activity levels of caspase 8 were much lower than those of the corresponding caspase 9 (*P*<0.05).

### Liposomal ASOs and Epi significantly affected Caco-2 cell cycle distribution

**Figure 6:**
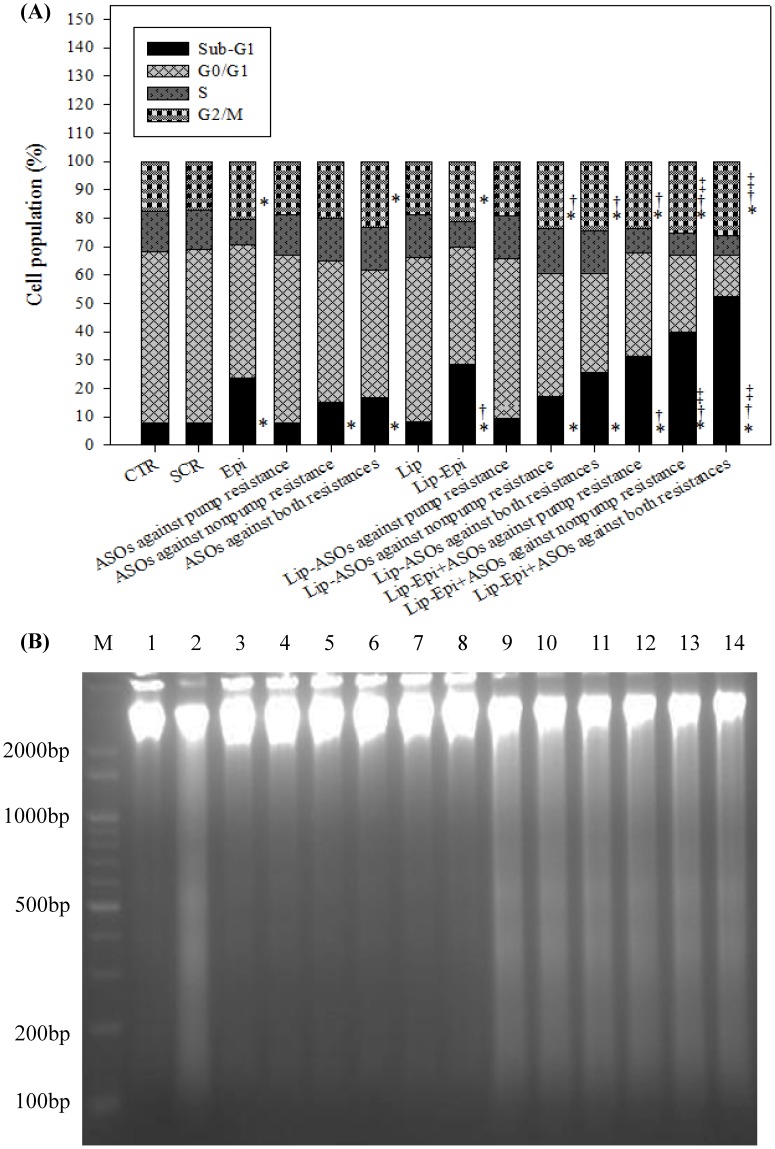
The effect of different treatments on the cell cycle distribution and DNA fragmentation in Caco-2 cells. (**A**) Analysis of cell number % of each cell cycle phase relative to total phases using flow cytometry in Caco-2 cells pretreated with different antisense formulations and/or Epi for 48 h. For example, sub-G1% is calculated as the percentage of the number of cells in the sub-G1 population relative to the number of total cells. Means ± S.D. from three independent experiments are shown. ^*^ *P*<0.05 compared to CTR; ^†^ *P*<0.05 compared to Epi; ^‡^ *P*<0.05 when liposomal formulation compared to its corresponding free formulation. (**B**) The DNA fragmentation effect of different treatments on Caco-2 cells for 48 h. DNA was isolated from Caco-2 cells and resolved by electrophoresis on 1.2% agarose gel. The gel was then visualized by SYBR safe staining. M: DNA marker; Lane 1: CTR; Lane 2: Epi; Lane 3: SCR; Lane 4: ASOs against pump resistance; Lane 5: ASOs against nonpump resistance; Lane 6: ASOs against both resistances; Lane 7: Lip; Lane 8: Lip-ASOs against pump resistance; Lane 9: Lip-Epi; Lane 10: Lip-ASOs against nonpump resistance; Lane 11: Lip-ASOs against both resistances; Lane 12: Lip-Epi+ASOs against pump resistance; Lane 13: Lip-Epi+ASOs against nonpump resistance; Lane 14: Lip-Epi+ASOs against both resistances.

Caco-2 cells after different treatments for 48 h showed a typical DNA pattern that represented sub-G1, G0/G1, S, and G2/M phases of the cell cycle. The percentages of apoptotic cells (sub-G1 phase) were significantly enhanced after cells were incubated with the treatments containing Epi, ASOs against pump resistance, nonpump resistance, or both resistaces in free or liposomal formulations for 48 h (Figure 6A). The cells treated with Lip-Epi+ASOs against both resistances exhibited the highest percentage of sub-G1 distribution (52.33±0.87%; *P*<0.05) among all the formulations, indicating that the treatment of this formulation induced more cells to undergo apoptosis.

### Liposomal ASOs and/or Epi significantly enhanced DNA fragmentation

DNA laddering is a molecular biological hallmark of apoptosis. During apoptosis, DNA unwound from histones is digested by endonuclease. The rest of the DNA organizes in a ladder pattern with multiples of approximately 100 to 200 bp subunits and can be separated by agarose gel electrophoresis. As shown in Figure 6B, DNA isolated from cells after treatment with Epi, Lip-Epi, and Lip-Epi plus ASOs against pump resistance, nonpump resistance, or both resistances formed a ladder pattern in DNA fragments. We further confirmed the DNA fragmentation phenomenon of Caco-2 cells triggered by apoptosis. Furthermore, we also observed that liposomal ASOs and Epi significantly induced chromatin condensation in Caco-2 cells using fluorescence microscopy (data not shown).

## Discussion

**Figure 7:**
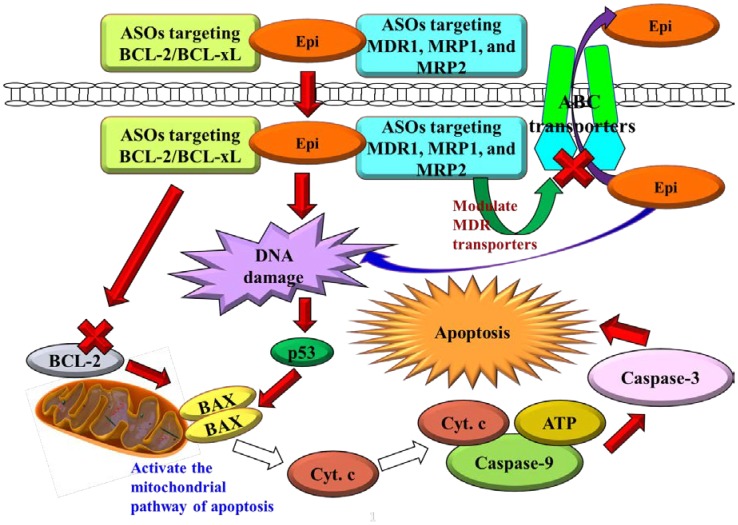
Proposed pathways for reversing pump and nonpump MDR in Caco-2 cells. Cyt. c, cytochrome c.

The combined use of ASOs with chemotherapy, such as Epi, is more active than ASO or Epi monotherapy, especially in cancer cell lines with MDR phenotype, such as Caco-2. P-gp, MRP1, MRP2, BCL-2, and BCL-xL are all related to the development of MDR and are present in Caco-2 cells [27]–[29]. Epi, an anticancer drug in the class of anthracycline, is a substrate of P-gp, MRP1, and MRP2, revealing that overcoming MDR may increase the therapeutic efficacy of Epi [8], [12]. Thus, exploring the synergistic effects of the combined therapy of Epi and ASOs against P-gp, MRP1, MRP2, and BCL-2/BCL-xL, may help us develop an effective strategy for multifunctional therapy of colon cancer. The proposed pathways for inhibiting MDR transporters and activating apoptosis in Caco-2 cells are shown in Figure 7.

The study employed cationic liposomes (100 nm) composed of DOTMA and DOPE core, coated by a PEG shell to carry ASOs and/or Epi. PEG may increase the half-life of this delivery system in systemic circulation by circumventing the reticuloendothelial system [30]. Furthermore, DOTMA is a monovalent cationic lipid, whereas DOPE is a neutral lipid used as a transfection enhancer in cationic liposome formulation. The PEGylated cationic liposomes might enhance the local accumulation of Epi and ASOs in the tumor tissue, possibly through the sustained release effect and enhanced permeability and retention (EPR) effect on the leaky tumor capillary fenestrations of cancer cells [31]–[33]. PEGylated stealth liposomes could be internalized into the cytoplasm or nuclei of cancer cells by endocytosis and membrane fusion to reduce the possibility of ASO degradation by nucleases after systemic administration [15]. Therefore, our delivery systems containing Epi and ASOs targeting MDR transporters (Lip-Epi+ASOs against pump resistance or both resistances) might increase the effective Epi concentration in cancer cells through antisense-mediated suppression of *hMDR1* promoter activity and MDR transporter expressions, resulting in the subsequent inhibition of Epi efflux, as shown in Figure 3 (decrease in the mRNA expression levels of MDR pump proteins) and Figure 4 (reduction in *hMDR1* promoter activity and increase in the intracellular accumulation of Epi). Moreover, ASOs against BCL-2/BCL-xL potentiated the apoptosis provoking effect of Epi, which activates the caspase-dependent apoptotic pathway, as demonstrated in Figure 5 (modulation on the mRNA expressions and activity levels of apoptosis-related proteins), Figure 6A (increase in sub-G1%), Figure 6B (DNA fragmentation), and Figure 2C (enhanced cytotoxicity).

In the current study, PEGylated liposomal Epi, used separately or together with ASOs significantly inhibited non-pump resistance by raising the mRNA expression levels of p53, which subsequently activated BAX and repressed BCL-2. These treatments triggered cell cycle arrest and significantly increased the expression and activity levels of caspase-3, -8, and -9. The activation of caspase 9 can be used to evaluate if the intrinsic or mitochondrial pathway is triggered, whereas the induction of caspase 8 can be used to determine if the extrinsic or death receptor pathway is provoked [34], [35]. Both pathways share the downstream effector caspases, such as caspase-3, -6, and -7 [12], [15], [36]. In the present study, incubating Caco-2 cells with Lip-Epi alone or combined with ASOs increased caspase 9 activities to higher levels than those of caspase 8, which suggested that an intrinsic apoptotic pathway through mitochondrial signaling was dominant in this apoptotic process. This finding was consistent with our previous study [5], [12] and other studies [37], [38]. However, the aforementioned treatments also mildly increased the expression and activity levels of caspase 8, which suggests that ASOs and Epi may also induce apoptosis through the extrinsic pathway. Further studies are needed to clarify the detailed process of the apoptosis pathway.

In this study, we found that the PEGylated liposomal ASOs targeting MDR1, MRP1, and MRP2 effectively reversed pump resistance. The encapsulated ASOs against pump resistance reduced the expressions of MDR transporters and decreased the function of these pump proteins, as signified by the higher intracellular Epi fluorescence than that of ASOs against nonpump resistance. This result seems to be reasonable because Yamanaka et al. (2006) found that the overexpression of BCL-2 and BCL-xL usually does not affect the influx and efflux of antineoplastic agents in cancer cells [11]. Consistently, ASOs against pump resistance genes in free or liposomal formulations seem to have no significant effect on the expression of BCL-2, which is a sensor for apoptotic stress. However, treating cells with anticancer drugs, such as doxorubicin elevated expression levels of BCL-2 in resistant tumor cells [39] and causes the development of multiple chemotherapy resistance [39], [40]. When cancer cells receive apoptotic stimuli, such as in the cases of Epi alone or combined treatment with ASOs against pump resistance, the expression of BCL-2 would arise to resist apoptotic induction from Epi. In contrast, ASOs targeting nonpump resistance decreased BCL-2 mRNA expression. Combined treatments of Epi and ASOs against nonpump resistance or both resistances resulted in significantly greater expression of BAX and BAX/BCL-2 ratio than those of Epi or Lip-Epi. The enhancement was highest in the case of Lip-Epi+ASOs against both resistances. The upregulation of BAX proteins increases chemosensitivity of tumor cells to various anticancer drugs [41]. A higher level of BAX/BCL-2 ratio indicates an increase in proapoptotic signal or a reduction in antiapoptotic expression, revealing critical intracellular targets for circumventing chemoresistance.

There was a correlation between the pump and nonpump resistance pathways. In addition to functioning as efflux pumps, MDR transporters also assist in escaping resistant cancer cells from apoptosis [13]. Accordingly, P-gp upregulation is related to BCL-2 or BCL-xL overexpression [42] and the suppression of caspase-8 and -3 [13]. Consistently, our study showed that Epi and ASOs targeting pump resistance or both resistance types decreased *hMDR1* promoter activity, inhibited MDR transporter expressions, but induced caspase-8, -9 and -3 expressions and activities in Caco-2 cells. The *hMDR1* promoter elements of 159 bp consist of an AP-1 site, a Y box, a CAAT site, and a GC box [25]. It has been reported that the binding of *c-fos* and *c-jun* with the AP-1 site positively regulates the *hMDR1* expression [43]. In addition, the *hMDR1* promoter elements may be regulated by the binding of the GC box to transcription factors such as Sp1 and the EGR family [24]. Furthermore, YB-1 and NF-Y binding sites located in the Y-box are essential for UV radiation to trigger the *hMDR1* promoter [25]. The induction or suppression of *hMDR1* promoter region by rifampicin or MDR inhibitors has been correlated with the increased or decreased levels of MDR1 mRNA expression in our previous studies [9], [12]. We thus suggest that liposomal Epi plus ASOs targeting MDR1, MRP1, MRP2 may bind to the specific site(s) on the *hMDR1* promoter elements and inhibit their activities. The inhibitory effect of Lip-Epi+ASOs against pump resistance or both resistances on the mRNA expression levels of MDR1 is in accordance with the transcriptional suppression of *hMDR1* promoter region. Thus, our study supports that suppression of MDR1 activates the intrinsic and extrinsic signaling pathways of apoptosis by inducing caspases. We believe that the inhibition of ATP-dependent efflux proteins such as P-gp and MRPs by Epi and ASOs against pump resistance or both resistances plays an important role in inducing caspase-dependent apoptosis.

In addition, we also found that PEGylated liposomal ASOs significantly intensified the intracellular uptake and chemosensitivity of Caco-2 cells to Epi, as well as potentiated Epi-induced apoptosis in mouse colon adenocarcinoma CT26 cells *in vitro* [33]. When PEGylated liposomal Epi and ASOs were administered by intravenous route, Epi had longer circulating half-life and greater area under the curve than in an Epi solution, as shown by an in *vivo* pharmacokinetic study using SD rats [33]. These Epi and ASOs delivery systems also substantially reduced tumor growth and significantly increased survival percentage of CT26-bearing Balb/c mice *in vivo* [33]. Our studies indicated that the PEGylated liposomal formulation of Epi and ASOs against both resistances demonstrated good cytotoxicity and/or antitumor efficacy in both human and mouse colon cancer.

In conclusion, this is the first report which indicates the reversing mechanisms of multiple ASOs not only in inducing apoptosis, but also in the inhibition of MDR transporters. Our findings provide a novel insight into the molecular mechanisms by which PEGylated DOTMA/DOPE liposomal ASOs targeting both resistance types enhance the chemosensitivity of colon cancer cells to Epi-provoked apoptosis through inhibiting MDR1, MRP1, and MRP2, as well as triggering intrinsic mitochondrial and extrinsic death receptor pathways. The complicated regulation of MDR highlights the necessity for a multifunctional approach using an effective delivery system, such as PEGylated liposomes, to carry epirubicin and ASOs as a potent nanomedicine for improving the clinical efficacy of chemotherapy.
