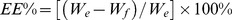# Correction: Reversing Multidrug Resistance in Caco-2 by Silencing MDR1, MRP1, MRP2, and BCL-2/BCL-xL Using Liposomal Antisense Oligonucleotides

**DOI:** 10.1371/journal.pone.0112604

**Published:** 2014-11-04

**Authors:** The PLOS ONE Staff

In the Materials and Methods section, there is an error in the first equation in the subsection “Encapsulation efficiency (EE%), size distribution, and zeta potential.” Please view the complete, correct equation here: